# Social contact patterns among employees in U.S. long-term care facilities during the COVID-19 pandemic, December 2020 to June 2021

**DOI:** 10.1186/s13104-023-06563-0

**Published:** 2023-10-26

**Authors:** Seth Zissette, Moses C. Kiti, Brady W. Bennett, Carol Y. Liu, Kristin N. Nelson, Alana Zelaya, Joseph T. Kellogg, Theodore M. Johnson II, Pam Clayton, Scott K. Fridkin, Saad B. Omer, Benjamin A. Lopman, Carly Adams

**Affiliations:** 1 Emory University, Atlanta, GA USA; 2 Georgia Health Care Association, Atlanta, GA USA; 3 Peter O’Donnell Jr. School of Public Health at UT Southwestern, Dallas, TX USA

**Keywords:** COVID-19, Long-term care facilities, Social contacts, Infection control

## Abstract

**Objective:**

We measured contact patterns using social contact diaries for 157 U.S. long-term care facility employees from December 2020 - June 2021. These data are crucial for analyzing mathematical transmission models and for informing healthcare setting infection control policy.

**Results:**

The median number of daily contacts was 10 (IQR 8–11). Household contacts were more likely partially masked than fully masked, more likely to involve physical contact, and longer in duration compared to facility contacts.

**Supplementary Information:**

The online version contains supplementary material available at 10.1186/s13104-023-06563-0.

## Introduction

Resident-care environments in nursing homes and assisted living facilities (collectively referred to as long-term care facilities, LTCFs) can allow rapid spread of infections among vulnerable populations as during the early COVID-19 pandemic. LTCFs recorded 150,000 COVID-19-related deaths from the pandemic onset through July 2022 [1, 2]. Facility lockdowns aimed at mitigating the spread of SARS-CoV-2 were implemented nationwide, with specific LTCF provisions on resident movement, group activities, and visitation [3]. Despite such measures, transmission continued, albeit at reduced levels. Staff necessarily enter and leave the facility but likely are a mechanism by which virus in the community may be introducing into LTCFs and cause subsequent transmission [4].

Measures of contact number and type are crucial inputs into mathematical models used to simulate outbreaks, inform transmission mitigation strategies, and guide COVID-19 response efforts [5, 6]. Data specific to LTCF populations are limited and differ from data from the general population due to frequent health care provision interactions and communal living. Quantification of LTCF-specific contact patterns may provide insight on COVID-19 transmission in LTCFs during the pandemic. Our study aimed to characterize the patterns of social contact and mixing of staff working in LTCFs in the United States using standardized social contact diaries.

## Main text

### Methods

The study team enrolled LTCF staff in Georgia through an online survey from December 2020 to June 2021. We partnered with the Georgia Health Care Association (GHCA), Georgia’s National Nursing Home COVID-19 Action Network (NNHCAN), and PruittHealth to disseminate recruitment flyers via newsletters, virtual meetings, and PruittHealth facility site visits. While the focus was on recruitment in the Southeastern United States, LTCFs from other regions participated resulting in a national convenience sample. Participants provided informed consent and completed an enrollment survey that collected information on sociodemographic characteristics, household structure, and job roles. Participants used a personalized email link to complete two consecutive days (48 h) of online electronic social contact diaries and received a gift card of $20 per day of contact diary completed ($40 maximum). Multiple daily contacts with a single individual counted as one contact, but were also tabulated by number of separate encounters and total duration of time spent. A contact was defined as any two-way conversation with an exchange of three or more words in the physical presence (i.e., someone close enough to touch) of another person. Contacts were classified as either non-physical or physical. A non-physical contact was one that did not involve touching; a physical contact involved touching, such as a handshake, or providing physical assistance. Contacts were recorded as not masked, fully masked (wearing a mask during the entire duration of encounters) or partially masked (wearing a mask for only a portion of the contact encounter). See Supplementary Material for the full data collection instruments.

Our primary analysis restricted the data to days where participants worked in the LTCF. We summarized the median number of contacts per person for the first day they completed a diary, stratified by selected characteristics. We also assessed the number of contacts participants had by the type of contact – LTCF resident, other LTCF staff, or household member. We additionally assessed the number of contacts by contact attributes, namely mask use, duration, location, and physical versus non-physical contact. Finally, a supplementary analysis was conducted using data from 67 participants who completed contact diaries on two work days for comparison. Within this subset, we were also able to analyze the frequency of unique contacts (a person listed as a contact on only one day) compared to repeated contacts (the same person listed as a contact on both days). Analyses were conducted in R v4.1.0. Emory University granted approval for human subjects’ research (IRB #STUDY00001344).

## Results

Of 211 total recruited participants from 196 facilities, 157 individuals from 157 unique facilities completed contact diaries for both days. 62 (39%) of these were from assisted living facilities and 95 (61%) were from nursing homes. Participants reported a total of 1,486 contacts. Most participants were between the ages of 30 and 49 (n = 145; 92%), identified as female (n = 80; 51%), non-Hispanic white (n = 121; 77%), and had a bachelor’s degree or higher (n = 133; 85%). 6 (4%) participants lived alone, 42 (27%) resided with a nuclear family unit (living with a partner and/or children only), and 107 (68%) lived with an extended family unit (living with extended family such as parents or siblings).

LTCF staff reported a mean of 9 and median of 10 daily contacts, with roughly half within the household (mean 4, median 5) and a mean of 5/median of 4 within the LTCF (Table 1). The mean and median numbers of contacts reported differed by age, household structure, job role, and month, but not by gender, education, or facility type. While job roles involving more patient care (e.g., clinical nurse assistant or registered nurse) reported more mean/median contacts (mean 10, median 12) compared to healthcare administration (mean 7, median 6), most of this was attributable to differences in the number of household contacts. Older individuals reported more mean/median contacts than younger individuals; participants aged 20–29 reported a mean and median of 5 contacts and participants aged 50 and older reported a mean of 13 and median of 15, with a steadily increasing trend for age groups between these two. Participants within extended family units reported more mean/median contacts (mean 10, median 10) than those living alone (mean 8, median 6) or in nuclear family units (mean 8, median 7), largely still driven by household contacts. Participants who completed contact diaries later in the study period (March 2021 to June 2021) reported a mean and median of 10 contacts, more than the 8 mean and 7 median contacts of those who completed earlier (December 2020 to February 2021), (IQR: 6–9). The supplementary analysis with data from two diary days did not differ substantially (see Table S1 in Supplementary Tables and Figures).

**Table 1:** Distribution of participant characteristics (n = 157) and the mean, median, and selected quantiles of contacts reported on first day worked during 2-day reporting period, December 2020 to June 2021

Participant characteristic	N = 157	Total			LTCF Resident			LTCF Staff			Household		
		Mean	Median	25th, 75th, & 90th percentiles	Mean	Median	25th, 75th, & 90th percentiles	Mean	Median	25th, 75th, & 90th percentiles	Mean	Median	25th, 75th, & 90th percentiles
**Total**	157 (100%)	9	10	8, 11, 12	3	3	2, 4, 5	2	1	1, 2, 3	4	5	3, 5, 6
**Gender**													
Female	80 (51%)	9	9	7, 11, 12	3	2	1, 3, 4	2	1	1, 2, 4	4	4	2, 5, 6
Male	77 (49%)	10	10	9, 11, 12	3	3	2, 4, 5	2	1	1, 2, 2	5	5	4, 6, 6
**Age group (years)**													
20–29	5 (3%)	5	5	1, 8, 9	1	1	1, 2, 3	2	2	1, 2, 3	2	0	0, 4, 4
30–39	62 (39%)	9	10	8, 11, 12	3	3	2, 4, 5	2	1	1, 2, 2	4	4	3, 5, 5
40–49	83 (53%)	10	10	9, 11, 12	3	3	2, 4, 4	2	1	1, 2, 3	5	5	4, 6 ,6
50+	7 (4%)	13	15	8, 19, 20	3	3	1, 5, 8	9	9	4, 14, 16	0	0	0, 0 ,0
**Race/Ethnicity**													
Asian	1 (1%)	4	4	4, 4, 4	4	4	4, 4, 4	0	0	0, 0, 0	0	0	0, 0, 0
Black	21 (13%)	7	10	4, 11, 11	2	2	1, 4, 4	2	1	1, 2, 4	3	4	0, 6, 6
Hispanic (All Races)	13 (8%)	9	10	10, 11, 13	3	3	3, 4, 4	2	1	1, 2, 3	4	5	4, 5, 6
Other	1 (1%)	5	5	5, 5, 5	0	0	0, 0, 0	4	4	4, 4, 4	0	0	0, 0, 0
White	121 (77%)	10	10	8, 11, 12	3	3	2, 4, 5	2	1	1, 2, 2	4	5	4, 5, 6
**Education**													
Bachelors or higher	133 (85%)	10	10	8, 11, 12	3	3	2, 4, 4	2	1	1, 2, 2	4	5	4, 5, 6
Less than Bachelors	24 (15%)	8	10	1, 12, 13	3	2	1, 5, 6	2	1	1, 3, 6	2	0	0, 6, 6
**Household Structure** ^**a**^													
Alone	6 (4%)	8	6	1, 17, 19	3	2	0, 6, 8	5	4	1, 9, 10	0	0	0, 0, 0
Nuclear	42 (27%)	8	7	6, 9, 11	2	2	1, 3, 3	3	1	1, 2, 5	2	3	0, 4, 4
Extended	107 (68%)	10	10	9, 11, 12	3	3	2, 4, 5	2	1	1, 2, 2	5	5	5, 6, 6
Other	2 (1%)	3	3	2, 4, 5	1	1	0, 1, 1	2	2	1, 3, 4	0	0	0, 0, 0
**Facility Type**													
Assisted Living Facility	62 (39%)	10	10	9, 11, 12	3	3	2, 4, 5	2	1	1, 2, 3	5	5	4, 6, 6
Nursing Home/	95 (61%)	9	10	7, 11, 13	3	3	1, 4, 5	2	1	1, 2, 4	4	4	2, 5, 6
Skilled Nursing Facility													
**Job Role**													
Advanced practice	8 (5%)	10	12	11, 12, 12	4	4	3, 4, 5	1	1	1, 1, 1	5	6	5, 6, 6
provider (APP)/Physician													
Clinical Nurse Assistant	20 (13%)	8	9	6, 11, 12	3	3	2, 5, 5	1	1	1, 1, 2	3	4	2, 5, 6
(CNA)													
Registered Nurse (RN)/	89 (57%)	10	10	8, 11, 12	3	3	2, 3, 4	2	1	1, 2, 2	5	5	4, 5, 6
Licensed Practical Nurse													
(LPN)													
Other patient-facing role^b^	21 (13%)	11	11	9, 11, 19	3	4	3, 4, 6	3	1	1, 1, 9	4	5	4, 5, 6
Environmental services	9 (6%)	10	11	10, 12, 13	3	3	3, 4, 5	3	2	1, 3, 4	4	5	4, 6, 6
worker													
Healthcare administration	10 (6%)	7	6	3, 12, 14	2	1	0, 2, 4	4	3	1, 5, 7	1	0	0, 1, 4
or non-patient care													
**Time of Data Collection**													
Dec. 2020 - Feb. 2021	35 (22%)	8	7	6, 9, 11	2	1	1, 2, 3	3	1	1, 2, 6	3	3	2, 4, 5
Mar. 2021 - Jun. 2021	122 (78%)	10	10	9, 11, 13	3	3	2, 4, 5	2	1	1, 2, 3	4	5	4, 6, 6

^a^Household structure was defined as a nuclear family unit if the participant lived with a partner and/or children only. Household structure was defined as an extended family unit if the participant also lived with extended family such as parents or siblings. Household structure was defined as “Other” if the participant lived primarily with a roommate or other non-family members

^b^Other patient-facing roles included physical therapist (PT), occupational therapist (OT), respiratory therapist, speech therapist, and social worker

**Fig. 1:**
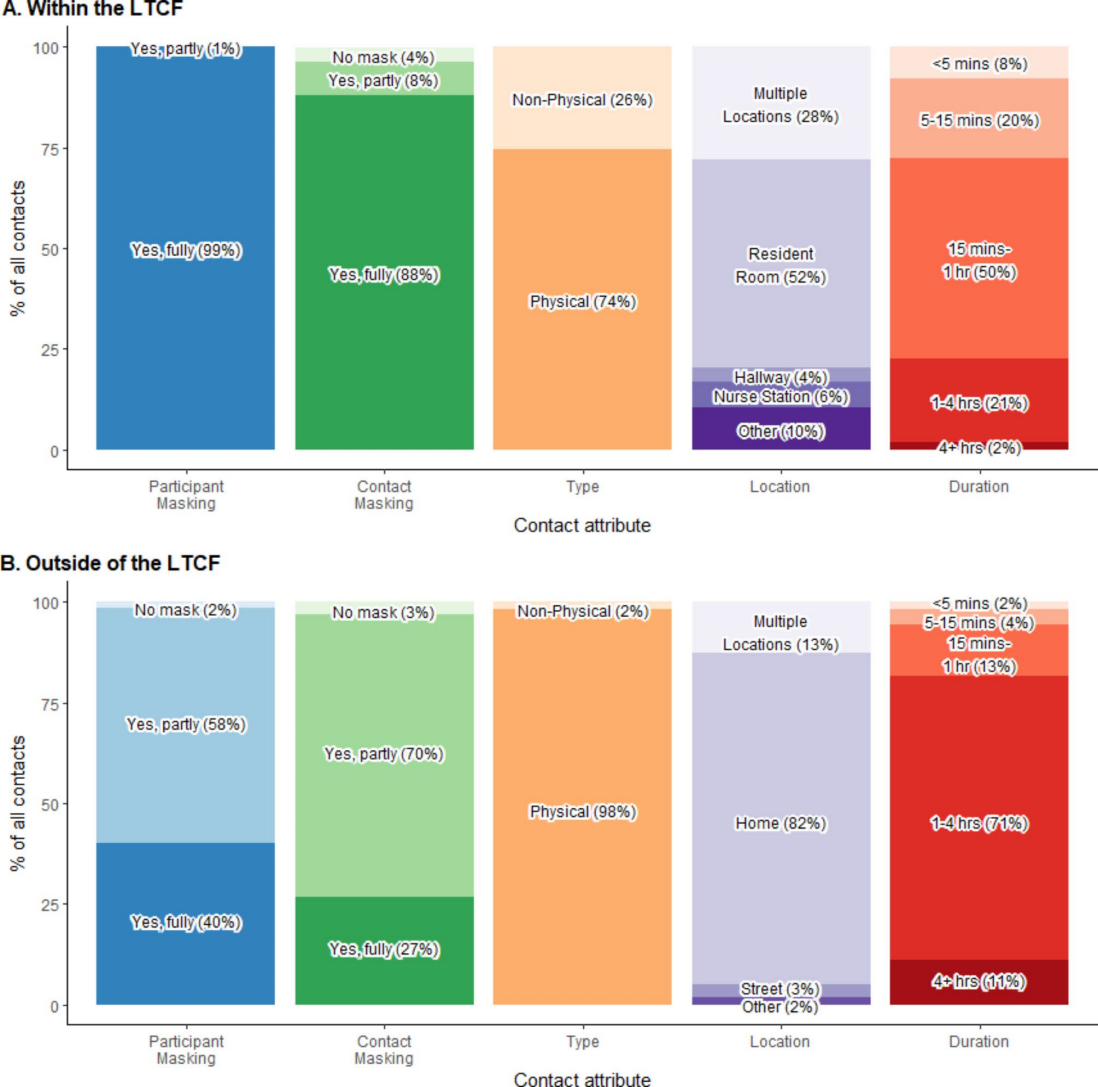
Distribution of contacts by attributes: whether the contact wore a mask (fully, partly, or not at all), duration of contact in minutes (mins) or hours (hrs), location, and type (physical or non-physical). Figure 1 A shows this distribution for 785 contacts reported by 156 participants over 156 diary-days. Figure 1B shows this distribution for 693 contacts reported by 135 participants over 135 diary-days

Participants reported differences in contact nature depending on whether contact occurred within or outside of the LTCF. Among the 1,486 contacts reported, 785 (53%) occurred within the LTCF and 693 (47%) occurred outside of the LTCF (Fig. 1). The most common location within the LTCF was inside a resident’s room (n = 405; 52% of contacts). The most common contact location outside the LTCF was at the participant’s home (n = 569; 82% of contacts). Within the LTCF, participants were fully masked for nearly all contacts (n = 784; 99%). Outside the LTCF, most contacts were partial masking (n = 405, 58%) with a small number of no mask contacts (n = 11, 2%). Individuals encountered by participants contacts within the LTCF were most frequently fully masked (n = 691; 88% of the time) while outside of the LTCF they were partially masked (n = 487; 70%). Contact duration was typically shorter within the LTCF, with 608 contact interactions (78%) lasting one hour or less, while 565 contacts (82%) outside of the LTCF lasted one hour or more. Participants indicated less physical contact in the LTCF (n = 584, 74%) compared to outside of the LTCF (n = 681; 97%). A supplementary analysis among those who completed two diary days on work days again did not differ substantially; however, using these data we could see that contacts within the LTCF were more likely to be unique (36%) compared to those outside of the LTCF (11% unique; see Figure S1 in Supplementary Tables and Figures).

## Discussion

LTCF staff in this study reported contact rates higher than the general community for the same time period [7–9]. Occupational contact rates in the LTCF setting were similar to those reported in previous work [10]. While study participants reported frequent use of precautions to prevent the spread of infection in line with standard LTCF infection control strategies while in the LTCF, their precautions outside of the facility were less stringent. Given the ease with which SARS-CoV-2 spreads asymptomatically, contacts outside the LTCF that were longer, involving more physical touch, and less likely to be masked bring a risk of introducing the virus into the LTCF particularly in the period prior to vaccination. Despite this, precautions for LTCF staff to take within their own households were not prominently featured in a national *Preventing the Spread of COVID-19* curriculum and may be important for future resources [11]. Because infection prevention measures (e.g., masking) are less likely to occur outside of LTCFs, stringent infection control protocols within LTCFs and early access to vaccination could help promote the safety of both LTCF residents and staff.

Despite limitations, these data may still provide a rationale for understanding and addressing contact patterns for LTCF staff working in a pandemic such as COVID-19. These data may be used to parameterize mathematical models that include staff contact with other staff, residents and community contacts. Even as management of COVID-19 improves and its burden on LTCFs decreases, understanding social contacts of LTCF staff more broadly and holistically will prove useful for future controlling of infectious diseases [12]. Further work into this area can continue to improve the lives of LTCF residents and the safety of their staff.

## Limitations

This study is limited by the small, non-representative sample in terms of participating staff, staff per facility, and type of job role. Participants were also from many different LTCFs, meaning that we cannot draw conclusions about contact networks within a given LTCF. Additionally, data are limited to self-reports over a timeframe of two days, translating to a single work shift for most LTCF staff. These data were likely susceptible to both measurement error, as participants completing contact diaries may likely to forget contacts or not report short-duration contacts [13], and social desirability bias [14], as some participants could have under-reported their true contact numbers due to social pressures around social distancing during the pandemic.

### Electronic supplementary material

Below is the link to the electronic supplementary material.

Supplementary Material 1

Supplementary Material 2

## Data Availability

The datasets used and analysed during the current study are available from the corresponding author on reasonable request.

## Abbreviations

**LTCF**: Long-term care facility

## References

[1] (2022). Trends in Number of COVID-19 Cases and Deaths in the US Reported to CDC, by Centers for Disease Control and Prevention, Territory S. https://covid.cdc.gov/covid-data-tracker.

[2] Centers for Medicare & Medicaid Services. (2022). COVID-19 Nursing Home Data. https://data.cms.gov/covid-19/covid-19-nursing-home-data/data. 26110197

[3] Centers for Medicare & Medicaid Services. (2020). “CMS Announces New Measures to Protect Nursing Home Residents from COVID-19.“ https://www.cms.gov/newsroom/press-releases/cms-announces-new-measures-protect-nursing-home-residents-covid-19.

[4] Adams C, Chamberlain A, Wang Y, Hazell M, Shah S, Holland DP, Khan F, Gandhi NR, Fridkin S, Zelner J, Lopman BA. (2022, May 19). The role of staff in transmission of SARS-CoV-2 in long-term care facilities. Epidemiology, 33(5), 669–677. 10.1097/ede.0000000000001510. 10.1097/EDE.0000000000001510 PMC9345519 35588282

[5] Liu CY, Berlin J, Kiti MC, Del Fava E, Grow A, Zagheni E, Melegaro A, Jenness SM, Omer SB, Lopman B, Nelson K (2021). Nov 1). Rapid Review of Social Contact patterns during the COVID-19 pandemic. Epidemiology.

[6] Padmanabhan R, Abed HS, Meskin N, Khattab T, Shraim M, Al-Hitmi MA (2021). Sep). A review of mathematical model-based scenario analysis and interventions for COVID-19. Comput Methods Programs Biomed.

[7] Brankston G, Merkley E, Fisman DN, Tuite AR, Poljak Z, Loewen PJ, Greer AL. (2021, 2021/11/08). Quantifying contact patterns in response to COVID-19 public health measures in Canada. BMC Public Health, 21(1), 2040. 10.1186/s12889-021-12080-1. 10.1186/s12889-021-12080-1 PMC8574152 34749676

[8] Feehan DM, Mahmud AS (2021). Quantifying population contact patterns in the United States during the COVID-19 pandemic. Nat Commun.

[9] Nelson KN, Siegler AJ, Sullivan PS, Bradley H, Hall E, Luisi N, Hipp-Ramsey P, Sanchez T, Shioda K, Lopman BA (2022). Jun 30). Nationally representative social contact patterns among U.S. adults, August 2020-April 2021. Epidemics.

[10] Assab R, Temime L (2016). 2016/08/09). The role of hand hygiene in controlling norovirus spread in nursing homes. BMC Infect Dis.

[11] Agency for Healthcare Research and Quality. (2022). Preventing the Spread of COVID-19. https://www.ahrq.gov/nursing-home/materials/index.html.

[12] Lee MH, Lee GA, Lee SH, Park Y-H. (2020). A systematic review on the causes of the transmission and control measures of outbreaks in long-term care facilities: back to basics of Infection control. PLoS ONE, 15(3), e0229911. 10.1371/journal.pone.0229911 PMC7064182 32155208

[13] Smieszek T, Burri EU, Scherzinger R, Scholz RW (2012). Collecting close-contact social mixing data with contact diaries: reporting errors and biases. Epidemiol Infect.

[14] Krumpal I (2013). Determinants of social desirability bias in sensitive surveys: a literature review. Qual Quant.

