# Supplementary material for: Social contact patterns among employees in U.S. long-term care facilities during the COVID-19 pandemic, December 2020 to June 2021
Source: BMC Res Notes. 2023 Oct 26;16:294. doi: 10.1186/s13104-023-06563-0 (PMC10604856; doi:10.1186/s13104-023-06563-0)
Supplement: Supplementary file 1 — Supplementary Material 1 [file 13104_2023_6563_MOESM1_ESM.pdf]

Nursing Home Mix Study

Enrollment Survey

Start of Block: Informed Consent

Q1 **CONSENT FOR PARTICIPATION IN A RESEARCH STUDY**
EMORY UNIVERSITY

Study Title: Comprehensively Profiling Social Mixing Patterns in Nursing Homes to Model COVID-19 Transmission (NursingHomeMix)

Principal Investigators: Benjamin Lopman, PhD MSc, Professor, Emory University Rollins School of Public Health
Mailing Address: 1518 Clifton Road, 4th Floor, Atlanta, GA 30322

Funding Source: Rollins School of Public Health (RSPH) Rapid COVID-19 Pilot Grant and R01 HS025987-03

Research Study Summary
We invite you to participate in a research study. The purpose of the study is to understand how nursing home staff interact with individuals within and outside of nursing homes. This information will be used to understand how COVID-19 and other diseases are transmitted. We are asking you to take part because you are an adult employee at one of our participating nursing homes. Participation is voluntary. You also can change your mind at any time. Whatever choice you make will not have any effect on your employment status.

Procedures
Study staff will be conducting two rounds of surveys (survey periods) in the nursing home: one in September-November 2020 and one in January-February 2021. Survey periods will each take place over 2 days (survey days): 1 day during the week (Monday - Friday) and 1 day during the weekend (Saturday - Sunday). You will only be asked to participate in the study on days that you work at the nursing home. If you agree to participate, you will be asked to complete the following: 1) a one-time online enrollment survey at the beginning of the study, and 2) online contact diaries for 1 to 2 workdays (24 hours each) during the two survey periods (up to 4 contact diaries in total). If you do not work on one or more of the survey days, you will not be asked to complete a contact diary for that day.

More information on each task is below:
1) The one-time online enrollment survey will begin immediately following this consent form and will collect information on your work schedule, who you live with, your marital status, and how

you commute to work, along with your contact information. This survey should take less than 10 minutes to complete.
2) The purpose of the contact diary is to collect data on your daily contacts, where the contact takes place, and for how long each contact lasts. A contact is defined as any two-way conversation with an exchange of three or more words in the physical presence (i.e., someone who is close enough to touch) of another person, and can be classified as either non-physical or physical. A non-physical (i.e., conversation only) contact is a contact that does not involve touching; a physical contact is a contact that does involve touching, such as a handshake, fist bump, hug, kiss, or providing physical assistance. In the contact diary, you will be asked to provide the following information on all contacts within and outside of the nursing home: who you had a contact with and for how long, specific information about each contact (such as age, gender, and whether they are a nursing home resident or staff), and other features of contacts. You will be coached on how to complete the contact diary. Contact diaries will be online and will be completed on your personal mobile device (by following a link to the diaries) or on a computer tablet provided by study staff (whichever you prefer). Each diary should take less than 30 minutes to complete.

Additionally, if you agree to participate, study staff will be given access to the baseline and follow-up surveys that you completed (or will complete) for the COPING (COVID-19 Prevention in Nursing Homes) Study. In these surveys, you were (or will be) asked about basic demographic information, details about prior testing for COVID-19, symptoms of recent illnesses, occupational activities, and exposures outside of the workplace.

Please note that for another, separate part of this study, we may ask you to wear a proximity sensor for a few days while working in the nursing home. This part of the study is also completely voluntary. Proximity sensors (or simply sensors) can be worn on the chest area (e.g., on a lanyard) and record data on who study participants have contact with and for how long. Your decision to participate in this contact diary portion of the study will not affect your eligibility for the sensor portion. If you agree to participate in this study (below), you will be agreeing to participate in the contact diary portion only.

Risks and Benefits: The possible risks or discomforts of the study are minimal and may include invasion of privacy (about the questions asked), unintentional release of data, and potential exposure to fomites (objects that may be contaminated with infectious agents, such as bacteria or viruses, and serve in their transmission). However, data protections, including deidentifying all data and storing data on password protected computers, will be used to ensure security and confidentiality of all data and findings. **Tablets will be thoroughly disinfected in between each use**. There are no immediate direct benefits to you for participating in this study,

however study findings will help us learn more about how to reduce the transmission of COVID-19 for everyone living and working in nursing homes in the future.

Costs and Compensation: There is no cost associated with participating in this study. In appreciation of your time, you will receive a $20 gift card for each contact diary you complete (up to $80).

Confidentiality: Your information from contact diaries and COPING study surveys will be kept fully confidential. Only the researchers involved in this study and those responsible for research oversight will have access to the information you provide. We will share it with others if you agree to it or when we have to do it because U.S. or State law requires it. When we publish the results of the research or talk about it in conferences, we will not use your name or other information that could identify you.

Contact Information If you have questions or if you have a research-related problem, please contact Dr. Benjamin A. Lopman at (404) 727-7485. If you have questions about your rights as a research participant, or you have complaints about this research, please call the Emory Institutional Review Board at (404) 712-0720 or email irb@emory.edu. You can reference the name of this study which is listed at the top of this form.

Please select your choice below. You may print a copy of this consent form for your records.
Clicking on the “Agree” button indicates that -
You are 18 years of age or older.
You have read and understood the above information.
You voluntarily agree to participate.

Q1 If you consent to the above, please click I Agree.

○ I agree

○ I disagree

Skip To: End of Survey If If you consent to the above, please click I Agree. = I disagree

End of Block: Informed Consent

Q2 Please enter your email address in the space below:

____

Q3 Please enter your phone number in the space below:

____

Q4 What is your marital status?

- ◯ Single or never married
- ◯ Married or domestic partnership
- ◯ Separated
- ◯ Divorced
- ◯ Widowed

Q5 Who do you live with? (Select all that apply)

- ☐ I live alone
- ☐ Spouse/significant other only
- ☐ Roommates
- ☐ Parents
- ☐ Children
- ☐ Siblings
- ☐ Other (Please specify) ____

Skip To: Q7 If Who do you live with? = I live alone

Q6 How many people are currently living or staying at your home?

INCLUDE:

- Yourself
- Everyone who has been living or staying at your home for more than 2 months
- Anyone staying with you who does not have another place to stay, *even if they have been with you for 2 months or less*

DO NOT INCLUDE: anyone who has been living somewhere else for more than 2 months (i.e. a college student living away or someone in the Armed Forces on deployment

▼ 2 (1) ... 10 (9)

Q7 What form of transit do you use to get to nursing home XXX? (Select all that apply)

- ☐ None
- ☐ Walking or biking
- ☐ Driving yourself
- ☐ Carpool with spouse/friends/coworkers
- ☐ Rideshare services
- ☐ Public transportation
- ☐ Other (please specify) ____

Q8 Which days of the week do you typically work at nursing home XXX? (Select all that apply)

- ☐ Mondays
- ☐ Tuesdays
- ☐ Wednesdays
- ☐ Thursdays
- ☐ Fridays
- ☐ Saturdays
- ☐ Sundays
- ☐ It typically varies

Q9 Which shift do you typically work at nursing home XXX?

- ○ Day shift
- ○ Night shift
- ○ It typically varies
- ○ Other (specify) ____

Nursing Home Mix Study

Social Contact Diary

Start of Block: About the Respondent

Q1 **Study Title**: Comprehensively Profiling Social Mixing Patterns in Nursing Homes to Model COVID-19 Transmission (NursingHomeMix)

Principal Investigator (the person who is responsible for this research): Benjamin Lopman, PhD MSc, Professor, Emory University Rollins School of School of Public Health
Mailing Address: 1518 Clifton Road, 4th Floor, Atlanta, GA 30322

Study Sponsor: Emory University and the National Institutes of Health (NIH)

Brief Introduction We would like you to record in the diary **every** person that you have **contact with** within and outside of the nursing home for your one or two designated days. This diary will help us learn how people interact within and outside of nursing homes, which helps us understand how respiratory diseases, such as COVID-19 and flu, may spread from one person to another. The diary should cover all contacts (both within and outside of the nursing home) for one or two full 24-hour days.

Definition of a contact: A contact is defined as:
· *Non-physical contact / conversation only* - a two-way conversation with three or more words in the physical presence of another person (close enough to touch) that does not involve touching.
· *Physical skin-to-skin contact* - a two-way conversation with three or more words in the physical presence of another person that involves touching (e.g., a handshake, fist bump, elbow bump, foot bump, hug, kiss, or providing physical assistance).

Write down every person that you come into contact with, regardless of whether the contact was long or short, and whether you know the person or not. Contacts made exclusively by phone should not be recorded. If you contact the same person several times in the course of the day, only record him/her once, but record the total time you spent with that person over the entire day.

After you have finished recording the diary, we suggest that you double check the diary entries to make sure you haven't missed any contacts or people. The order in which you write down your contact persons is not important. However, we suggest using a chronological order, starting from when you met the person for the first time during your assigned day, and then adding anyone else that you might remember as you went through your daily activities.

Q2 Please enter your phone number in the space below:

____

Q3 Please enter your email address in the space below:

____

Q4 Is this your first or second contact diary for this survey period?

- ○ First
- ○ Second

Q5 What part of the week is it?

- ○ Weekday (Monday – Friday)
- ○ Weekend (Saturday – Sunday)

Q6 How many people did you come into contact (as defined above) with today?

____

End of Block: About the Respondent

Start of Block: Social Contact Questions (Loop & Merge) for Resident Contacts

Q7 Were any of these contacts nursing home residents?

◯ Yes

◯ No

If No, skip to Q10

Q8 How many nursing home residents did you have contact with today?

▼ 1 (1) ... > 20 (21)

Number of columns in table below =

Q8 How many nursing home residents did you have contact with today?

Q9 Please complete the table below for all residents you had contact with today.

- You can enter in this information by clicking on a box and typing in your information.
- You can place a check mark in boxes by clicking on the screen.

**Floor:**_______		**Floor:**______		**Floor:**_______		**Floor:**_______	
**Unit:**_______		**Unit:**_______		**Unit:**_______		**Unit:**_______	
**Place of Contact** (select all)		**Place of Contact** (select all)		**Place of Contact** (select all)		**Place of Contact** (select all)	
	Within resident's room		Within resident's room		Within resident's room		Within resident's room
	Outside resident's room		Outside resident's room		Outside resident's room		Outside resident's room
**Estimated Age Range**		**Estimated Age Range**		**Estimated Age Range**		**Estimated Age Range**	
	< 40 years		< 40 years		< 40 years		< 40 years
	40 – 49 years		40 – 49 years		40 – 49 years		40 – 49 years
	50 – 59 years		50 – 59 years		50 – 59 years		50 – 59 years
	60 – 69 years		60 – 69 years		60 – 69 years		60 – 69 years
	70 – 79 years		70 – 79 years		70 – 79 years		70 – 79 years
	80 – 89 years		80 – 89 years		80 – 89 years		80 – 89 years
	≥ 90 years		≥ 90 years		≥ 90 years		≥ 90 years
**Gender**		**Gender**		**Gender**		**Gender**	
	Male		Male		Male		Male
	Female		Female		Female		Female
	Other: _____________		Other: _______________		Other: _______________		Other: _______________
**Did you touch this resident?**		**Did you touch this resident?**		**Did you touch this resident?**		**Did you touch this resident?**	
	Yes		Yes		Yes		Yes
	No		No		No		No
**Care Activities** (select all)		**Care Activities** (select all)		**Care Activities** (select all)		**Care Activities** (select all)	
	Hygiene (oral, bathing, etc.)		Hygiene (oral, bathing, etc.)		Hygiene (oral, bathing, etc.)		Hygiene (oral, bathing, etc.)
	Medical administration		Medical administration		Medical administration		Medical administration

	Dressing resident		Dressing resident		Dressing resident		Dressing resident
	Transfer (bed to chair, etc.)		Transfer (bed to chair, etc.)		Transfer (bed to chair, etc.)		Transfer (bed to chair, etc.)
	Wound care		Wound care		Wound care		Wound care
	Vent / trach care		Vent / trach care		Vent / trach care		Vent / trach care
	Device care – urine / central catheter		Device care – urine / central catheter		Device care – urine / central catheter		Device care – urine / central catheter
	Stool cleanup		Stool cleanup		Stool cleanup		Stool cleanup
	Linen change		Linen change		Linen change		Linen change
	PT/OT		PT/OT		PT/OT		PT/OT
	Glucometer		Glucometer		Glucometer		Glucometer
	Vital signs		Vital signs		Vital signs		Vital signs
	Nutrition/feeding – PEG		Nutrition/feeding – PEG		Nutrition/feeding – PEG		Nutrition/feeding – PEG
	Other: _______________		Other: _______________		Other: _______________		Other: _______________
	No care activities		No care activities		No care activities		No care activities
**Typical frequency of contact with this resident**		**Typical frequency of contact with this resident**		**Typical frequency of contact with this resident**		**Typical frequency of contact with this resident**	
	Multiple times per day		Multiple times per day		Multiple times per day		Multiple times per day
	Daily or almost daily		Daily or almost daily		Daily or almost daily		Daily or almost daily
	Once or twice per week		Once or twice per week		Once or twice per week		Once or twice per week
	Once or twice per month		Once or twice per month		Once or twice per month		Once or twice per month
	Never met before		Never met before		Never met before		Never met before
**Total time spent with resident during entire day**		**Total time spent with resident during entire day**		**Total time spent with resident during entire day**		**Total time spent with resident during entire day**	
	Less than 5 minutes		Less than 5 minutes		Less than 5 minutes		Less than 5 minutes
	5 to 15 minutes		5 to 15 minutes		5 to 15 minutes		5 to 15 minutes
	16 to 59 minutes		16 to 59 minutes		16 to 59 minutes		16 to 59 minutes
	1 to 4 hours		1 to 4 hours		1 to 4 hours		1 to 4 hours
	More than 4 hours		More than 4 hours		More than 4 hours		More than 4 hours

---

Q10 You have entered information for ${Q8/ChoiceTextEntryValue} nursing home resident contacts. Do you want to enter information for any additional nursing home resident contacts?

○ Yes

○ No

Display this Question if:

You have entered information for ${Q8/ChoiceTextEntryValue} nursing home resident contacts. Do you want to enter... = Yes

Q11 How many additional nursing resident contacts do you want to enter?

▼ 1 (1) ... > 20 (21)

Questions repeat for additional nursing home contacts, starting with question 9

End of Block: Social Contact Questions (Loop & Merge) for Resident Contacts

---

Start of Block: Social Contact Questions (Loop & Merge) for Non-resident Contacts

You are evaluating person ${lm://CurrentLoopNumber} out of ${lm://TotalLoops}

Q12 Please enter a brief description of this contact (e.g. man wearing red shirt, first and/or last name, initials, etc.), whatever helps you remember this contact (this information will not be used for any other purpose):

____

Q13 If this is your second contact diary, did you have contact with this person on the first day?

○ Yes

○ No

○ This is day 1 contact diary for me.

Q14 What is the contact's gender?

- ○ Male
- ○ Female
- ○ Other (please describe) ____
- ○ I don't know

Q15 Please choose the age range of this contact. Estimate if you are not sure.

- ○ Less than 6 months old
- ○ 6 to 11 months old
- ○ 1 to 4 years old
- ○ 5 to 9 years old
- ○ 10 to 19 years old
- ○ 20 to 29 years old
- ○ 30 to 39 years old
- ○ 40 to 49 years old
- ○ 50 to 59 years old
- ○ 60 to 69 years old
- ○ 70 to 79 years old
- ○ 80 years old and older

Q16 Did this encounter occur within or outside of nursing home XXX?

◯ Within

◯ Outside

If Outside, skip to Q19

Q17 Was this person a nursing home staff, visitor, or other?

◯ Staff

◯ Visitor

◯ Other (specify) ________________________________

If Visitor or Other, skip to Q20

Q18 What type of staff was this person?

- ◯ Registered Nurse (RN)
- ◯ License Vocational Nurse (LVN) or Licensed Practical Nurse (LPN)
- ◯ Certified Nursing Assistant (CNA)
- ◯ Physical Therapist (PT) or Occupational Therapist (OT)
- ◯ Respiratory therapist
- ◯ Physician
- ◯ Social worker
- ◯ Healthcare administration or non-patient care
- ◯ Environmental service worker
- ◯ Other (specify) ____
- ◯ Unknown

Skip to Q20

Display This Question only if:

Q16 Did this encounter occur within or outside of nursing home XXX? = Outside

Q19 Where did you have contact with this person?

- ☐ Home
- ☐ School/College
- ☐ Transport/hub
- ☐ Leisure
- ☐ Store/Mall
- ☐ Street
- ☐ Healthcare setting (e.g. hospital, clinic)
- ☐ Place of worship
- ☐ Playground
- ☐ Another person's home
- ☐ Other ____

Q20 Did you touch this person?

- ○ Yes
- ○ No

If No, skip to Q22

Q21 Please select the type of physical contact below (choose all that apply).

- ☐ Handshake
- ☐ Hug
- ☐ Fist Bump
- ☐ Elbow Bump
- ☐ Foot Bump
- ☐ High five
- ☐ Other (specify): ________________

Q22 How often do you have contact with this person, in general?

- ○ Multiple times per day
- ○ Daily or almost daily
- ○ About once or twice a week
- ○ About once or twice a month
- ○ Less than once a month
- ○ Never met before

Q23 What was the total time you spent with this person during the entire day?

- ◯ Less than 5 minutes
- ◯ Between 5 to 15 minutes
- ◯ Between 15 minutes to 1 hour
- ◯ Between 1 hour to 4 hours
- ◯ More than 4 hours

End of Block: Social Contact Questions (Loop & Merge)

Start of Block: Block 2

Q24 Did you attend any gatherings today (e.g. meetings, grocery store, hospitals, public performances, movie theaters, etc.) where you had contact with a group of people?
As a reminder, a contact is defined as:
· ***Non-physical contact / conversation only*** - a two-way conversation with three or more words in the physical presence of another person (close enough to touch) that does not involve touching.
· ***Physical skin-to-skin contact*** - a two-way conversation with three or more words in the physical presence of another person that involves touching (e.g., a handshake, fist bump, elbow bump, foot bump, hug, kiss, or providing physical assistance).

- ◯ Yes
- ◯ No

If No, skip to Q28

Display this Question if:

Q24 Did you attend any gatherings today (e.g. meetings, grocery store, hospitals, public performances, movie theaters, etc.) where you had contact with a group of people? = Yes

Q25 Did this gathering take place at work in nursing home XXX or outside of work?

- ◯ At work
- ◯ Outside of work

Display this Question if:

Q24 Did you attend any gatherings today (e.g. meetings, grocery store, hospitals, public performances, movie theaters, etc.) where you had contact with a group of people? = Yes

Q26 Please list the approximate number of people who attended the *largest* gathering today (at work or outside of work) in the space below.

____

Display this Question if:

Q24 Did you attend any gatherings today (e.g. meetings, grocery store, hospitals, public performances, movie theaters, etc.) where you had contact with a group of people? = Yes

Q27 Please list the approximate length (in minutes) of the *longest* gathering (at work or outside of work) in the space below.

____

End of Block: Block 2

Start of Block: Block 3

Q28 You have entered information for ${Q4/ChoiceTextEntryValue} contacts outside of work. Do you want to enter information for any additional contacts?

○ Yes

○ No

Display this Question if:

You have entered information for ${q://QID2/ChoiceTextEntryValue} contacts outside of work. Do you want to enter... = Yes

Q29 How many additional contacts do you want to enter?

▼ 1 (1) ... > 10 (11)

End of Block: Block 3

Start of Block: Social Contact Questions ADDITIONAL (Loop & Merge 2)

Q30

You are evaluating person ${lm://CurrentLoopNumber} out of ${lm://TotalLoops}

Questions repeat for additional contacts, starting with question 12
