# Supplementary material for: Social contact patterns among employees in U.S. long-term care facilities during the COVID-19 pandemic, December 2020 to June 2021
Source: BMC Res Notes. 2023 Oct 26;16:294. doi: 10.1186/s13104-023-06563-0 (PMC10604856; doi:10.1186/s13104-023-06563-0)
Supplement: Supplementary file 2 — Supplementary Material 2 [file 13104_2023_6563_MOESM2_ESM.pdf]

Table S1. Distribution of participant characteristics (n = 67) for those who completed contact diaries on two work days and the mean, median, and selected quantiles of daily contacts reported over the two study days overall and by type of contact, December 2020 to June 2021.

Participant characteristic	N=67	Total			LTCF Resident			LTCF Staff			Household		
		Mean	Median	25th, 75th, & 90th percentiles	Mean	Median	25th, 75th, & 90th percentiles	Mean	Median	25th, 75th, & 90th percentiles	Mean	Median	25th, 75th, & 90th percentiles
**Total**	67 (100%)	10	10	8, 12, 13	3	3	2, 4, 5	2	1	1, 2, 5	4	5	0, 5, 6
**Gender**													
Female	33 (49%)	10	10	7, 11, 15	3	2	1, 4, 6	3	1	1, 2, 10	3	4	0, 5, 5
Male	34 (51%)	10	10	9, 12, 13	3	3	2, 4, 5	2	1	1, 2, 3	4	5	4, 6, 6
**Age group (years)**													
20-29	3 (4%)	2	1	1, 2, 4	1	1	0, 1, 1	1	1	0, 2, 3	0	0	0, 0, 0
30-39	26 (39%)	8	10	7, 11, 12	3	2	2, 4, 5	2	1	1, 2, 2	3	4	0, 5, 5
40-49	32 (48%)	11	10	9, 12, 14	3	3	2, 4, 5	2	1	1, 2, 3	5	5	5, 6, 6
50+	6 (9%)	12	13	4, 19, 21	4	3	0, 7, 10	8	6	2, 13, 18	0	0	0, 0, 1
**Race/Ethnicity**													
Asian	1 (1%)	4	4	4, 5, 5	4	4	4, 5, 5	0	0	0, 0, 0	0	0	0, 0, 0
Black	10 (15%)	7	6	2, 11, 13	2	2	1, 4, 5	2	2	1, 3, 4	2	0	0, 6, 6
Hispanic (All Races)	5 (7%)	8	10	3, 12, 12	3	4	0, 5, 5	2	1	1, 1, 2	3	4	0, 5, 6
White	51 (76%)	11	10	9, 11, 14	3	3	2, 4, 6	3	1	1, 2, 5	4	5	4, 5, 6
**Education**													
Bachelors or higher	52 (78%)	10	10	9, 11, 13	3	3	2, 4, 5	2	1	1, 2, 4	4	5	4, 5, 6
Less than Bachelors	15 (22%)	8	8	1, 13, 14	3	2	1, 5, 6	2	2	1, 3, 5	2	0	0, 5, 6
**Household Structure[a]**													
Alone	6 (9%)	8	2	1, 12, 19	3	2	0, 7, 10	5	2	1, 8, 12	0	0	0, 0, 0
Nuclear	15 (22%)	9	8	4, 11, 15	2	1	0, 3, 4	4	1	1, 4, 12	2	0	0, 3, 5
Extended	45 (67%)	10	10	9, 12, 13	3	3	2, 4, 5	2	1	1, 2, 3	5	5	4, 6, 6
Other	1 (1%)	1	1	1, 1, 1	1	1	1, 1, 1	0	0	0, 0, 0	0	0	0, 0, 0

**Facility Type**													
Assisted Living Facility	23 (34%)	11	11	10, 12, 13	4	4	3, 5, 5	1	1	1, 2, 3	5	5	5, 6, 6
Nursing Home/ Skilled Nursing Facility	44 (66%)	9	9	5, 11, 14	3	2	1, 3, 5	3	1	1, 2, 8	3	4	0, 5, 5
**Job Role**													
Advanced practice provider (APP)/Physician	5 (7%)	10	12	10, 12, 12	3	4	3, 4, 4	1	1	1, 1, 1	5	6	5, 6, 6
Clinical Nurse Assistant (CNA)	5 (7%)	5	4	1, 8, 12	2	2	1, 3, 6	1	1	1, 1, 2	2	0	0, 3, 5
Registered Nurse (RN)/ Licensed Practical Nurse (LPN)	24 (36%)	10	10	9, 10, 12	3	2	2, 3, 4	2	1	1, 2, 3	4	5	4, 5, 5
Other patient-facing role[b]	18 (27%)	11	10	9, 12, 16	4	4	3, 5, 6	2	1	1, 1, 7	4	5	0, 6, 6
Environmental services worker	6 (9%)	12	12	11, 13, 14	4	4	3, 5, 5	3	2	2, 3, 3	5	6	4, 6, 7
Healthcare administration or non-patient care	9 (13%)	7	7	2, 11, 14	2	1	0, 3, 4	4	2	1, 5, 9	1	0	0, 4, 4
**Time of Data Collection**													
Dec. 2020 - Feb. 2021	12 (18%)	9	9	4, 11, 16	2	1	0, 2, 3	4	1	1, 7, 12	2	1	0, 4, 5
Mar. 2021 - Jun. 2021	55 (82%)	10	10	9, 12, 13	3	3	2, 4, 5	2	1	1, 2, 3	4	5	4, 5, 6

[a]Household structure was defined as a nuclear family unit if the participant lived with a partner and/or children only. Household structure was defined as an extended family unit if the participant also lived with extended family such as parents or siblings. Household structure was defined as "Other" if the participant lived primarily with a roommate or other non-family members.

[b]Other patient-facing roles included physical therapist (PT), occupational therapist (OT), respiratory therapist, speech therapist, and social worker.

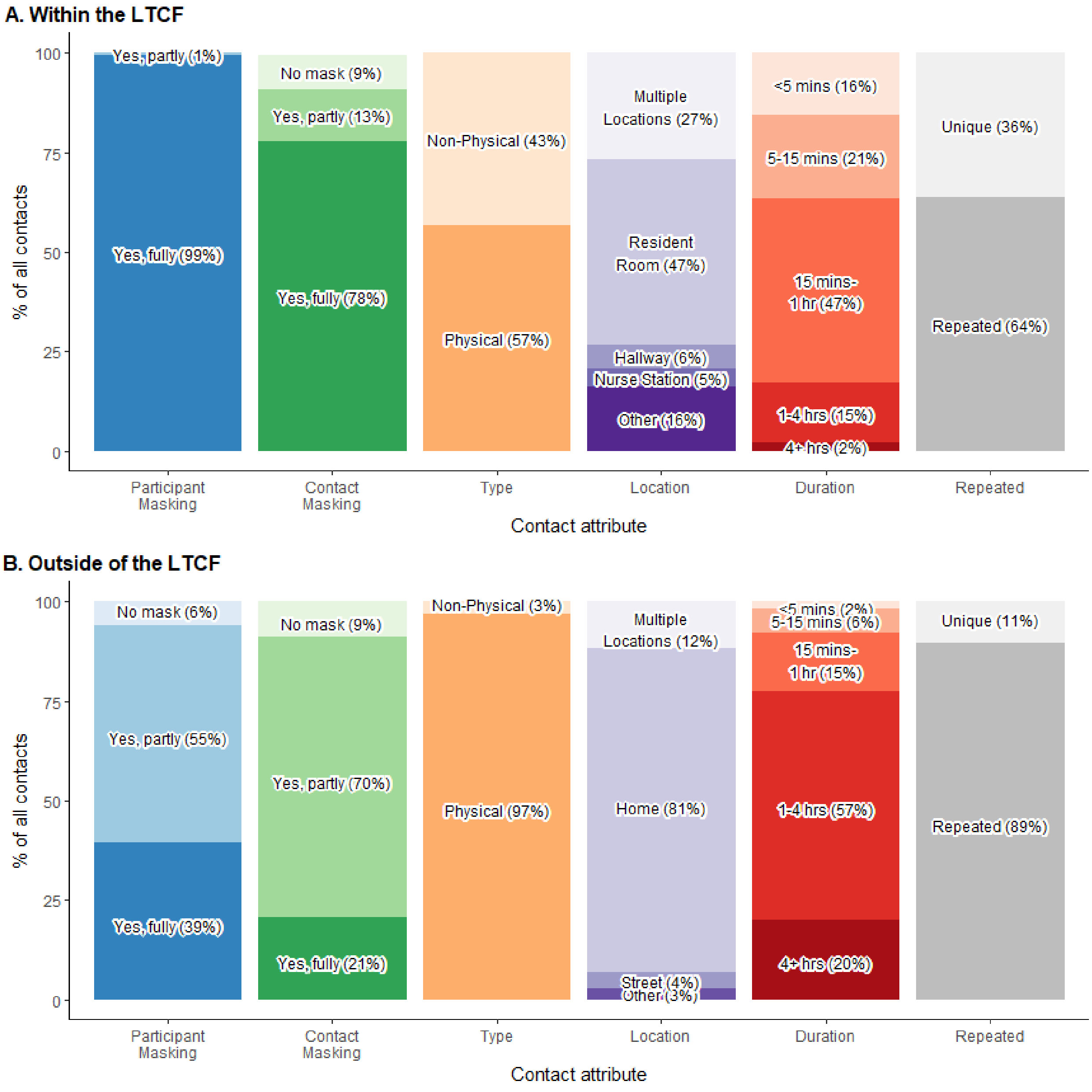

Figure S1. Distribution of contacts by attributes: whether the contact wore a mask (fully, partly, or not at all), duration of contact in minutes (mins) or hours (hrs), location, and type (physical or non-physical). Fig. S1A shows this distribution for 748 contacts reported by 67 participants over 134 diary-days. Fig. S1B shows this distribution for 546 contacts reported by 52 participants over 134 diary-days.
